# NID1, a new regulator of EMT required for metastasis and chemoresistance of ovarian cancer cells

**DOI:** 10.18632/oncotarget.16145

**Published:** 2017-03-13

**Authors:** Ya Zhou, Yuanyuan Zhu, Xiaoyan Fan, Chundong Zhang, Yitao Wang, Lian Zhang, Huan Zhang, Tao Wen, Kaina Zhang, Xiao Huo, Xue Jiang, Youquan Bu, Ying Zhang

**Affiliations:** ^1^ Department of Biochemistry and Molecular Biology, Chongqing Medical University, Chongqing 400016, China; ^2^ Molecular Medicine and Cancer Research Center, Chongqing Medical University, Chongqing 400016, China; ^3^ College of Pharmacy, Chongqing Medical University, Chongqing 400016, China; ^4^ First Clinical College, Chongqing Medical University, Chongqing 400016, China; ^5^ College of Biomedical Engineering, Chongqing Medical University, Chongqing 400016, China

**Keywords:** ovarian cancer, NID1, EMT, metastasis, chemoresistance

## Abstract

Nidogen-1 (NID1) has been identified as a novel candidate diagnostic biomarker of ovarian cancer in our previous study. Nevertheless, the role of NID1 in the pathogenesis of ovarian cancer is unclear. In the present study, we demonstrated that NID1 was a mesenchymal associated gene and its high expression was significantly correlated with shorter overall survival of ovarian cancer patients. The ectopic expression of NID1 in OVCAR-3 cells revealed a epithelial-mesenchymal transition (EMT) phenotype accompanied by enhancement of motility, invasiveness and cisplatin resistance, whereas the knockdown of NID1 was sufficient to convert HEY cells into epithelial phenotype with decreased capability of motility, invasiveness and cisplatin resistance. Mechanistic studies disclosed that NID1 activated ERK/MAPK signaling pathway to promote EMT. Collectively, our findings have uncovered the molecular mechanisms of NID1 in promoting ovarian cancer metastasis and chemoresistance, and provide a rationale for the therapeutic potential of NID1 suppression in ovarian cancer.

## INTRODUCTION

Ovarian cancer is the most lethal gynecological malignancy worldwide [1]. In China, it showed an increasing trend in incidence and mortality for ovarian cancer, with an estimated 52 100 new cases and 22 500 deaths from this disease in 2015 [2]. The lethality of ovarian cancer is mainly attributed to the fact that most ovarian cancers are undiagnosed until advanced stages. Despite the evolution of surgical techniques and chemotherapy regimens, most patients with advanced-stage ovarian cancer experience a relapse, with a median progression-free survival of only 18 months [3]. Ovarian cancer to data is still a poorly understood disease with an extremely poor prognosis. To provide insight that will enable the development of new therapeutics, it is of paramount importance to elucidate the molecular mechanisms regarding the migratory and invasive properties of ovarian cancer cells.

Epithelial-mesenchymal transition (EMT) is a complex multi-step process whereby epithelial cells lose their polarity and acquire the migratory characteristics of mesenchymal cells. Concurrent with a loss of epithelial markers (E-cadherin, α-catenin, γ-catenin, *etc*.), cells undergoing EMT gain mesenchymal cell markers (N-cadherin, vimentin, fibronectin, *etc*.) [4]. There is a greater flexibility in this transitional process, during which cells linger in intermediary stages and they frequently undergo a partial EMT program, rather than a complete EMT program [5]. EMT has been recognized to be pivotal for embryonic development, tissue fibrosis and cancer progression. Moreover, EMT is an initiating event in metastasis of epithelial cancers. Genetic and epigenetic alterations can induce migration and invasion of carcinoma cells, possibly through an EMT. At secondary sites, these cells form a new carcinoma through a mesenchymal-epithelial transition (MET) [6]. Epithelial ovarian cancers constitute 90% of ovarian cancers, thus EMT is also essential for the acquisition of the metastatic property in ovarian cancer [7, 8].

Basement membrane is a thin layer of highly specialized extracellular matrix, which underline cells and separate cells from and connect them to their interstitial matrix [9]. Cancer cells must cross basement membrane barriers to spread and then seed at new sites by recruiting a basement membrane-containing vascular supply to expand, therefore the basement membrane is closely linked with cancer invasion and metastasis [10]. The main components of basement membrane include collagen IV, laminins, perlecan and nidogens. Of these, nidogens are linkers connecting collagen IV and laminin networks, further stabilizing three-dimensional structure of the basement membrane [11]. The nidogen family in human consists of two members, nidogen-1 (NID1) and nidogen-2 (NID2). In the previous ovarian cancer relevant proteomic study, we found NID1 was a candidate diagnostic biomarker for ovarian cancer; it is elevated in the peripheral blood of ovarian cancer patients compared to normal controls [12, 13]. To date, few studies have reported on the non-structural role of NID1. NID1 enhanced cell spreading in HEK293T cells [14], or promoted skin wound healing [15, 16]. In addition, inhibition of NID1 reduced the migration and invasion of ETV5 overexpressing endometrial cancer cells [17]. However, its detailed function in ovarian cancer remains unknown. Taking a close relationship between cancer metastasis, EMT and basement membrane into consideration, we wondered whether NID1 involved in ovarian cancer EMT and thus metastasis. In this study, we present the first evidence that NID1 promotes the migration, invasion and chemoresistance of ovarian cancer cells via EMT and its molecular mechanism involves the activation of ERK/MAPK signaling.

## RESULTS

### NID1 is a mesenchymal associated gene

**Figure 1:**
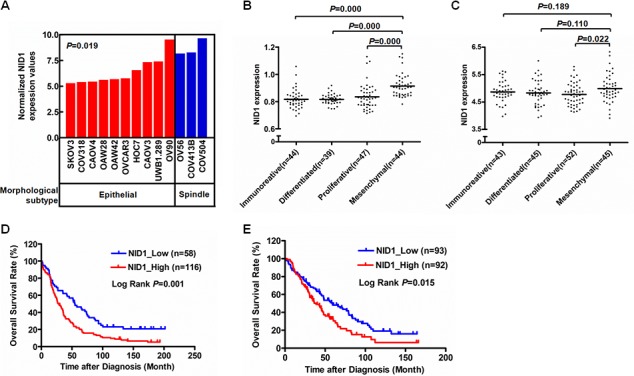
NID1 was a meshenchymal associated gene **(A)** NID1 expression level in indicated ovarian cancer cell lines with two morphological subtypes (10 epithelial and 3 spindle). The ovarian cancer cell line gene expression data processed by 2log normalization was extracted from the Helleman cohort (GSE53418). Statistical analysis was done using two-sided independent Student's t test. **(B-C)** NID1 expression level in ovarian cancer tissues with four molecular subtypes (“immunoreative”, “differentiated”, “proliferative” and “mesenchymal”). The gene expression datasets of 174 and 185 high-grade serous ovarian cancer tissue processed by 2log normalization were extracted from the Mayo Clinic cohort (GSE53963) (B) and the Bonome cohort (GSE26712) (C), respectively. Statistical analysis was done using two-sided independent Student's t test. **(D-E)** Prognostic significance of NID1 in ovarian cancer. Kaplan-Meier plot of overall survival of patients with high-grade serous ovarian cancer were obtained from the Mayo Clinic cohort (GSE53963) (D)and the Bonome cohort (GSE26712) (E), respectively. The survival curves were compared using the log-rank test. *P*-values were two-sided and *P*<0.05 was considered statistically significant.

Epithelial ovarian cancer is a highly heterogeneous disease and the publicly available ovarian cancer cell lines undoubtedly present distinct morphologic characteristics. Beaufort and colleagues characterized a series of ovarian cancer cell lines into a spindle subtype and an epithelial subtype, based on hierarchical clustering using differentially expressed genes [18]. Of these, NID1 showed high expression in the spindle subtype, rather than the epithelial subtype (*P*<0.05) (Figure 1A). Additionally, gene expression analysis of high-grade serous ovarian cancer tissues were performed and then designated four molecular subtypes (“immunoreactive”, “differentiated”, “proliferative”, and “mesenchymal”), from the Australian Ovarian Cancer Study and the Cancer Genome Atlas Research Network study [19, 20]. The four molecular subtypes were then applied to the Mayo Clinic cohort [21] and the Bonome cohort [22]. For both, expression of NID1 was significantly elevated in the “mesenchymal” subtype than that in the other three subtypes (*P*<0.05) (Figure 1B and 1C). Moreover, high expression of NID1 was correlated with shorter overall survival of ovarian cancer patients (*P*<0.05) (Figure 1D and 1E). The relevance of its high expression and the Spindle as well as the “mesnencymal” subtype shows that NID1 probably imparts EMT-prone phenotype to ovarian cancer cells.

### NID1 regulates the phenotype of ovarian cancer cells through EMT process

**Figure 2:**
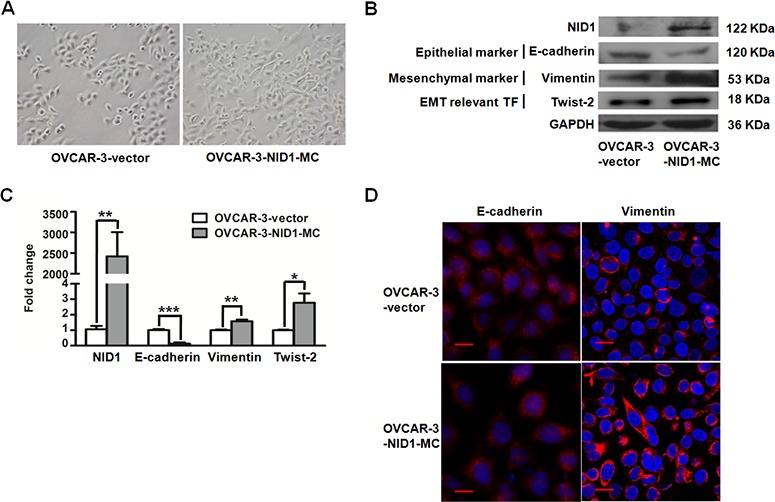
Expression of NID1 in OVCAR-3 cells induced partial EMT process **(A)** Representative microscopic views of OVCAR-3-vector and OVCAR-3-NID1-MC cells (200×magnification). **(B)** Western blot for NID1, E-cadherin, Vimentin and Twist-2 in the indicated cells, with GAPDH as the loading control. **(C)** Quantitative RT-PCR for NID1, E-cadherin, Vimentin and Twist-2 in the indicated cells, with GAPDH as the quantitative control. Two independent experiments were performed in triplicates (Two-sided independent Student's t test, *P<0.05, ^*^P<0.01, ^**^*P<0.001). **(D)** Immunofluorescent microscopic images of E-cadherin (red) and Vimentin (red) and in the indicated cells. Nuclear was stained with 4,6-diamidino-2-phenylindole (blue) (scale bar = 20 μm).

To obtain further insights into the EMT-promoting capability of NID1 in ovarian cancer, we first generated the stable OVCAR-3 monoclone with ectopic expression of NID1. The OVCAR-3-NID1-MC cells showed markedly enhanced NID1 mRNA (*P*<0.01; Figure 2C) and NID1 protein levels (Figure 2B), with a shift from cobblestone-like morphology to a spindle shape (Figure 2A and Figure 2D). Furthermore, OVCAR-3-NID1-MC cells demonstrated a decrease in the epithelial marker E-cadherin but an increase in mesenchymal marker Vimentin and the EMT transcription factor Twist-2, compared to OVCAR-3-vector cells (Figure 2B and Figure 2C). Consistent with this, immunofluoresence detection showed the coherent change of Vimentin, whereas no change of E-cadherin, in OVCAR-3-NID1-MC cells (Figure 2D). It appears stable expression of NID1 in OVCAR-3 cells cause a partial EMT process.

**Figure 3:**
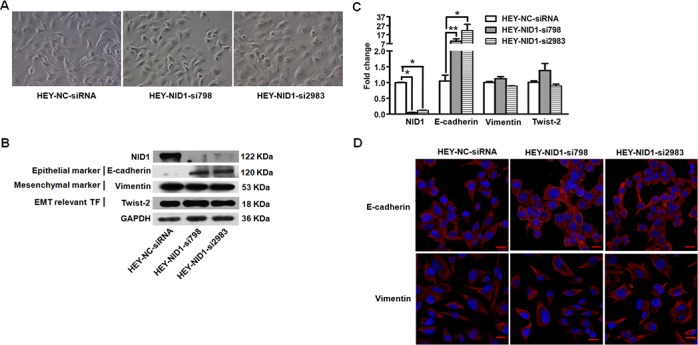
NID1 silencing in HEY cells induced partial MET process **(A)** Representative microscopic views of HEY-NC-siRNA, HEY-NID1-si798 and HEY-NID1-si2983 cells (200×magnification). **(B)** Western blot for NID1, E-cadherin, Vimentin and Twist-2 in the indicated cells, with GAPDH as the loading control. **(C)** Quantitative RT-PCR for NID1, E-cadherin, Vimentin and Twist-2 in the indicated cells, with GAPDH as the quantitative control. Two independent experiments were performed in triplicates (Two-sided independent Student's t test, *P<0.05, ^*^P<0.01). **(D)** Immunofluorescent microscopic images of E-cadherin (red) and Vimentin (red) and in the indicated cells. Nuclear was stained with 4,6-diamidino-2-phenylindole (blue) (scale bar = 20 μm).

Similar results were observed in NID1-silencing HEY cells. The HEY-NID1-si798 and HEY-NID1-si2983 cells showed significantly reduced NID1 mRNA (*P*<0.05; Figure 3C) and NID1 protein levels (Figure 3B). NID1 knockdown resulted in a dramatic reversion towards the epithelial phenotype, which altered cell morphology from a spindle shape to a cobblestone-like shape (Figure 3A and Figure 3D). Re-expression of the epithelial marker E-cadherin was detected in HEY cells after NID1 silencing, along with unchanged levels of the mesenchymal marker Vimentin and the EMT transcription factor Twist-2 (Figure 3B). Immunofluoresence analyses showed that NID1 knockdown changed E-cadherin in its cellular distribution not its expression level. Compared to its cytosolic localization in HEY-NC-siRNA cells, E-cadherin alternatively appeared on the plasma membrane/cell junction in both of HEY-NID1-si798 and HEY-NID1-si2983 cells (Figure 3D). It indicates that NID1 silencing induces a partial MET process in ovarian cancer cells. This finding is in accordance to the aforementioned clues from ectopic NID1 expression relevant experiments. The present results lend support to the EMT-promoting role of NID1 in ovarian cancer.

### ERK/MAPK signaling pathway is involved in EMT process induced by NID1

**Figure 4:**
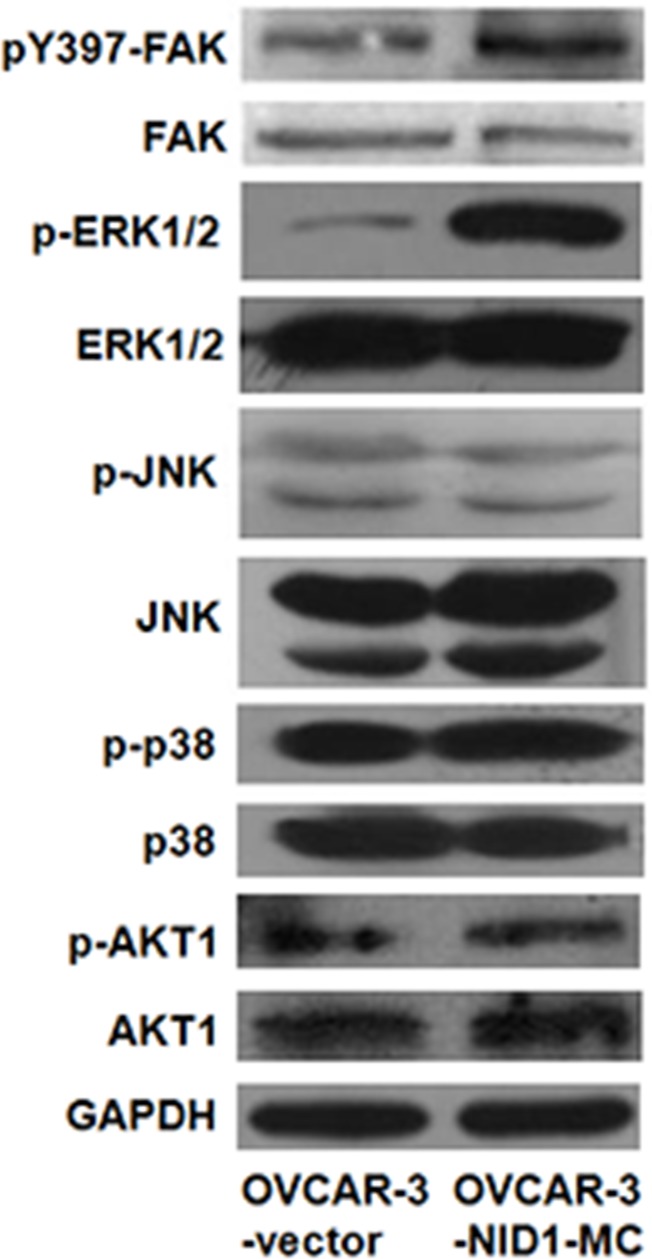
Effect of NID1 on signaling pathways Western blot for FAK, ERK1/2, JUK, p38 and AKT1 and their corresponding activation in total cell lysates of OVCAR-3-vector and OVCAR-3-NID1-MC cells. GAPDH served as an internal control of protein loading.

**Figure 5:**
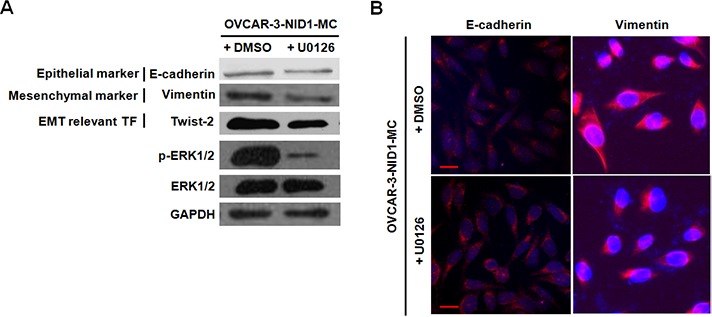
The NID1-induced expression of EMT relevant markers was regressed in the presence of U0126 (a MEK1 inhibitor) **(A)** Western blot for E-cadherin, Vimentin, Twist-2, phoshpo-ERK1/2 and total ERK1/2 in total cell lysates of OVCAR-3-NID1-MC cells after U0126 treatment. GAPDH served as an internal control of protein loading. **(B)** Immunofluorescent microscopic images of E-cadherin (red) and Vimentin (red) in OVCAR-3-NID1-MC cells after U0126 treatment. Nuclear was stained with 4,6-diamidino-2-phenylindole (blue) (scale bar = 20 μm).

Previous studies have reported that MAPK pathways and PI3K/AKT pathway involve in cancer cell migration and invasion via upregulating Twist in a variety of human cancers [23–26]. Therefore, we ascertained whether these pathways function in the EMT-promoting role of NID1or not. As shown in Figure 4, ectopic expression of NID1 increased the abundance of phosphorylated (activated) ERK1/2, whereas it didn't change the levels of phosphorylated (activated) JNK, phosphorylated (activated) p38 and phosphorylated (activated) AKT1. Conversely, NID1 knockdown slightly reduced the abundance of phosphorylated ERK1/2 (Supplementary Figure 1). Thus, we hypothesized that the EMT-promoting function of NID1 in ovarian cancer cells might result from the activation of ERK/MAPK pathway. To address this issue, OVCAR-2-NID1-MC cells were treated with a MEK1 inhibitor (U0126), which significantly reduced the level of phosphorylated ERK1/2. Importantly, U0126 abrogated the NID1-induced expression of Vimentin and Twist-2 afterwards, without alteration of E-cadherin expression (Figure 5A and 5B). These findings reveal that NID1 impinges on ERK/MAPK signaling pathway to foster the EMT of ovarian cancer cells.

### NID1 exacerbates ovarian cancer cell migration and invasion

**Figure 6:**
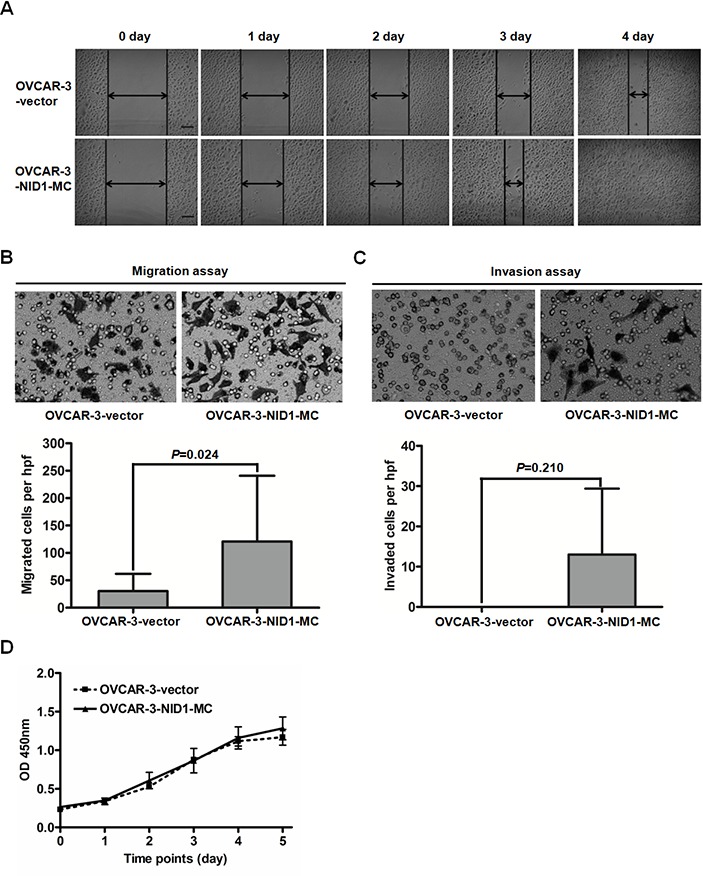
Expression of NID1 in OVCAR-3 cells facilitated cell migration and invasion **(A)** Representative scraping wounds formed by OVCAR-3-vector and OVCAR-3-NID1-MC cells were measured at 0, 1, 2, 3 and 4 days after wounding (scale bar = 100 μm). **(B)** Representative microscopic views of migratory cells from OVCAR-3-vector and OVCAR-3-NID1-MC cells were photographed at 100×magnification (upper). Five fields of the indicated cells were enumerated (lower). **(C)** Representative microscopic views of invaded cells from OVCAR-3-vector and OVCAR-3-NID1-MC cells were photographed at 100×magnification (upper). Five fields of the indicated cells were enumerated (lower). Means and S.Ds are shown from three independent experiments performed in duplicates. **(D)** Proliferation curves of OVCAR-3-vector and OVCAR-3-NID1-MC cells were monitored every 24 h, over 5 days. Means and S.Ds at each time point are shown from three independent experiments performed in triplicates. Statistical analysis was done using two-sided paired Student's t test or independent Student's t test. *P*<0.05 was considered statistically significant.

EMT is currently considered as a key event for tumor metastasis, we next determined the effect of NID1 on ovarian cancer cell migration and invasion. Matrigel migration/invasion assays showed that migratory and invasive OVCAR-3-NID1-MC cells were both more than migratory and invasive OVCAR-3-vector cells (*P*=0.024; Figure 6B and P=0.210; Figure 6C). Concordantly, the wound-healing capacity of OVCAR-3-NID1-MC cells was dramatically greater than that of OVCAR-3-vector cells (Figure 6A). NID1 overexpression didn't significantly affect the proliferation rate of OVCAR-3 cells (*P*=0.591; Figure 6D).

**Figure 7:**
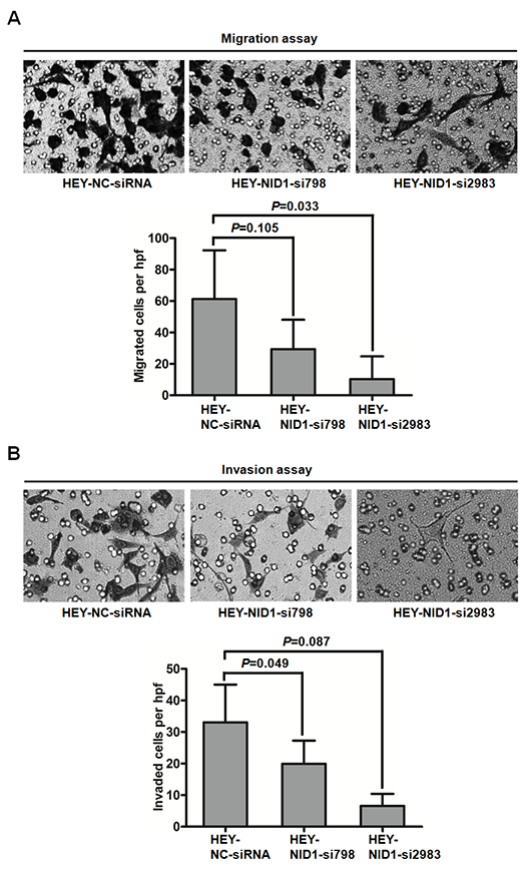
NID1 silencing in HEY cells suppressed cell migration and invasion **(A)** Representative microscopic views of migratory cells from HEY-NC-siRNA, HEY-NID1-si798 and HEY-NID1-si2983 cells were photographed at 100×magnification (upper). Five fields of the indicated cells were enumerated (lower). **(B)** Representative microscopic views of invaded cells from HEY-NC-siRNA, HEY-NID1-si798 and HEY-NID1-si2983 cells were photographed at 100×magnification (upper). Five fields of the indicated cells were enumerated (lower). Means and S.Ds are shown from two independent experiments performed in duplicates. Statistical analysis was done using paired Student's t test or two-sided independent Student's t test. *P*<0.05 was considered statistically significant.

In contrast, NID1 silencing in HEY cells reduced cell migration and invasiveness. Compared to HEY-NC-siRNA cells, HEY-NID1-si798 and HEY-NID1-si2983 cells both exhibited weaker migration capacity (*P*=0.105, *P*=0.033; Figure 7A), as well as invasion capacity (*P*=0.049, *P*=0.087; Figure 7B). Together, these data suggest a positive role of NID1 in the motility and invasiveness of ovarian cancer cells associated with partial EMT process.

### NID1 promotes the cisplatin-based resistance of ovarian cancer cells

**Figure 8:**
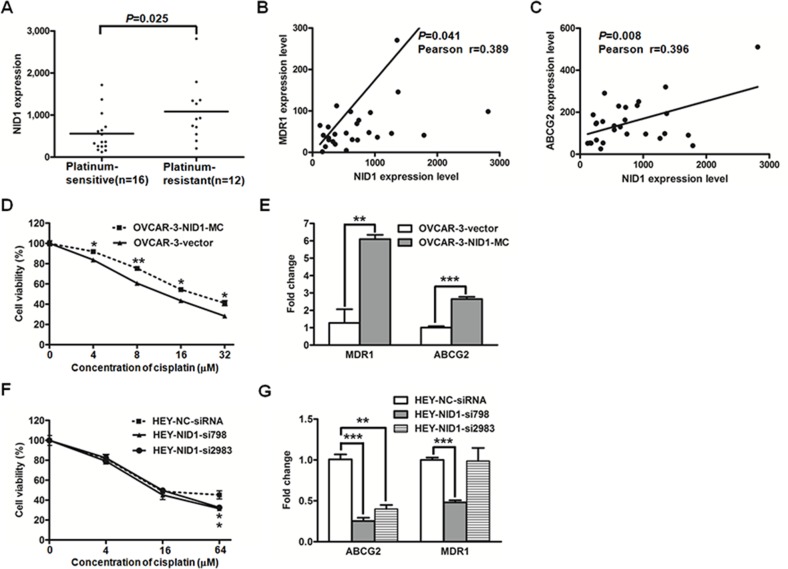
The gene expression datasets of 28 high-grade serous ovarian cancer tissue processed by 2log normalization was extracted from the Koti group (GSE51373) NID1 expression level was indicated in the platinum-sensitive group and the platinum-resistant group **(A)** Correlations between the expression levels of NID1 and MDR1 **(B)** or ABCG2 **(C)** were evaluated with the Pearson correlation test. **(D)** The cell viability of OVCAR-3-vector and OVCAR-3-NID1-MC cells treated with various concentrations of cisplatin was measured by CCK-8 assay. **(E)** Quantitative RT-PCR for MDR1 and ABCG2 in the indicated cells, with GAPDH as the quantitative control. **(F)** The cell viability of HEY-NC-siRNA, HEY-NID1-si798 and HEY-NID1-si2983 cells treated with various concentrations of cisplatin was measured by CCK-8 assay. **(G)** Quantitative RTPCR for MDR1 and ABCG2 in the indicated cells, with GAPDH as the quantitative control. Three independent experiments were performed in triplicates. Statistical analysis was done using twosided independent Student's t test. *P<0.05, **P<0.01, ***P<0.001.

Taking the relevance of EMT with chemoresistance of cancer cells into consideration, we further investigated the effects of NID1 on cisplatin-based resistance in ovarian cancer cells. Koti and colleages performed the whole transcriptome profiling with 28 high-grade serous ovarian cancer tissues, which were divided into the platinum-sensitive group and the platinum-resistant group [27]. The NID1 level was remarkably higher in the resistant group than that in the sensitive group (*P*=0.025; Figure 8A), and its level was positively correlated with the well-characterized drug efflux transporters MDR1 (*P*=0.041, Figure 8B) and ABCG2 (*P*=0.008; Figure 8C), respectively.

These results described above suggested that NID1 might contribute to chemoresistance. To test this premise, we examined cisplatin-sensitivity of OVCAR-3-vector cells and OVCAR-3-NID1-MC cells. Compared to OVCAR-3-vector cells, OVCAR-3-NID1-MC cells exhibited higher cisplatin resistance (*P*<0.05; Figure 8D) and correspondingly higher expression levels of MDR1 and ABCG2 (*P*<0.01; Figure 8E). Furthermore, the cisplatin-sensitivity of NID1-silencing HEY cells were detected as well. HEY-NC-siRNA cells showed slightly higher cisplatin resistance (*P*<0.05; Figure 8F) and higher expression levels of MDR1 and ABCG2 (*P*<0.05; Figure 8G), relative to NID1-silencing HEY cells.

## DISCUSSION

We previously demonstrated that NID1 was elevated in the peripheral blood of ovarian cancer patients compared to normal controls [12, 13]. Its abnormal expression level probably alter the integral organization of basement membrane and its functions. In the present study, NID1 was also indicated as a mesenchymal associated protein, based on its higher expression in spindle- and mesenchymal-subtypes (Figure 1). Furthermore, NID1 promoted the EMT process of ovarian cancer cells whereby they lose their epithelial characteristics and acquire certain mesenchymal properties (Figure 2). In classical EMT, cancer cells undergoing EMT present a loss of epithelial markers (E-cadherin, *etc*.) and a gain of mesenchymal cell markers (Vimentin, *etc*.). Nevertheless, the expression patterns of EMT markers do not follow this model in this study. The immunofluoresence detection didn't show the alteration of E-cadherin in NID1-overexpressed OVCAR-3 cells. Accordingly, Vimentin didn't change obviously in NID1-silencing HEY cells. They all suggest ovarian cancer cells undergo a partial EMT process. A partial EMT state has been noted in many developmental, wound healing, fibrosis, and cancer processes, with the existence of intermediate hybrid epithelial and mesnechymal phenotype [5]. The complexity of EMT in ovarian cancer cells probably stems from their heterogeneous nature. Epithelial ovarian cancer is the predominant form of ovarian cancer, comprising several histological subtypes based on tumor cell morphology. The four major histological subtypes include serous, mucinous, clear cell, and endometrioid. Of these, the serous type is the most common, originating from fallopian tube epithelium. OVCAR-3 cells and HEY cells are of the serous ovarian cancer subtype. Previous reports indicate that intermediate states of EMT display the highest plasticity, and thus represent an appropriate stage within which to induce or reverse EMT [28]. However, OVCAR-3 cells and HEY cells present epithelial phenotype and mesenchymal phenotype, respectively. The effect of NID1 is limited, therefore these ovarian cancer cells separately retain partial intrinsic characteristics during the transition to their opposite phenotype.

Twist-1 and Twist-2 are bHLH transcription factors responsible for EMT. Twist-1 has been reported in a role in MAPK pathway to mediate EMT [23, 24]. However, our data suggested that EMT induced by NID1 was associated with aberrant activation of ERK/MAPK pathway which then functions on Twist-2, not Twist-1 (Figure 2B and Figure 5A). Integrins are heterodimeric cell surface glycoproteins consisting of α and β subunits and mediate the attachment of cells to the extracellular matrix (ECM). The binding of integrins and the ECM leads to the recruitment of focal adhesion kinase (FAK) to the newly formed focal adhesion sites. Subsequently, FAK acts as a phosphorylation-regulated signaling scaffold, which activates downstream effectors (such as ERK/MAPK pathway, JNK/MAPK pathway and PI3K/AKT pathway) and then results in cell migration and invasion [29]. As a critical component in basement membrane, NID1 was able to bind with integrin α3β1 [30]. Therefore, we examined if integrin-FAK signaling involved in the activation of ERK/MAPK pathway by NID1. Herein we demonstrated that integrin α3 was also expressed in OVCAR-3-vector cells and OVCAR-3-NID1-MC cells (Supplementary Figure 2). Ectopic expression of NID1 enhanced the phosphorylation of FAK at Y397 (Figure 4). Furthermore, treatment of PF573228 (an FAK inhibitor) remarkably reduced the level of phosphorylation Y397 in FAK as well as that of phosphorylated ERK1/2, but it hardly changed the expressions of EMT-relevant markers (Supplementary Figure 3). All these points raised the possibility that the diminution of integrin-FAK signaling provoked other downstream pathways which compensated the effects of ERK/MAPK inactivation. In this context, FAK is inapplicable as an drug target in ovarian cancer treatment.

The EMT has been implicated in two of the most important process responsible for cancer-related mortality: progression to distant metastatic disease and acquisition of therapeutic resistance [31]. We demonstrated NID1 promoted ovarian cancer cell migration and invasion (Figure 6 and Figure 7). There are few reports with respect to the pathological role of NID1 in cancer progression. In agreement with our observation, inhibition of NID1 reduced the migration and invasion of ETV5-overexpressed endometrial cancer cells [17]. In addition, we found NID1 favored cisplatin-based resistance of ovarian cancer, with refractory patients having the higher expression level of NID1, NID1-overexpressed cells having higher cisplatin resistance and NID1-silencing cells having weaker cisplatin resistance (Figure 8A, Figure 8D and Figure 8F). The occurrence of cisplatin-resistance is correlated with a plethora of molecular mechanisms, including enhancing cytotoxic agents extrusion by ATP-binding cassette (ABC) transporters [32]. Herein we proposed that NID1 exacerbated resistance of ovarian cancer cells via upregulating the expression of MDR1 and ABCG2 (Figure 8E and Figure 8G), two well-known ABC transporters. Besides NID1, several regulators, such as HPIP, NANOG and FOXM1, have been reported to promote EMT of ovarian cancer cells and then confer them drug resistance, involving PI3K/AKT pathway, STAT3 pathway, *etc* [33–36]. The aforementioned results implicated that NID1-overexpressed ovarian cancer cells potentially exhibited cancer stem cell-like characteristics which imparts the metastatic and chemoresistant advantage to cells. As an example, the expression level of CD44 (one ovarian cancer stem cell marker) was increased in NID1-overexpressed OVCAR-3 cells but decreased in NID1-depleted HEY cells (Supplementary Figure 4). Recent evidence has highlighted a link between EMT and cancer stem cells that favor metastasis and therapeutic resistance of tumors, and the subtypes of cancer stem cells that display therapeutic resistance and phenotypic plasticity may be promising therapeutic targets [37]. In further work, we would focus on these issues.

In summary, our study shows that NID1 is a mesenchymal associated gene and is significantly correlated with poor prognosis of ovarian cancer. Moreover, NID1 plays a critical role in ovarian cancer cell migration, invasion and chemoresistance by partial EMT process. The underlying mechanism involves, at least in part, the activation of ERK/MAPK signaling pathway. Thus, NID1 may represent a candidate prognostic indicator and a potential therapeutic target of ovarian cancer.

## MATERIALS AND METHODS

### Cell culture, construction of stable cell lines and siRNA transfection

The human ovarian papillary serous adenocarcinoma cell line HEY was obtained from Shanghai Genechem (Shanghai, China). The human ovarian papillary serous adenocarcinoma cell line OVCAR-3 was donated by Dr. Huhua Ling (Department of Obstetrics and Gynecology, First Affiliated Hospital, Chongqing Medical University). Cells were cultured in RPMI 1640 medium (Invitrogen, Carlsbad, CA, USA) supplemented with with 10% fetal bovine serum (Invitrogen, Carlsbad, CA, USA), streptomycin (100 μg/mL) and penicillin (100 IU/ml). All cells were maintained in a humidified incubator at 37°C with 5% CO_2_.

OVCAR-3 cells were selected to generate cells with stable NID1 overexpression. Transfection of OVCAR-3 cells with 4.0 μg control plasmid (GV144) (Shanghai Genechem, Shanghai, China) or NID1 expression vector (NID1-GV144) (Shanghai Genechem, Shanghai, China) was performed using Lipofectamine 2000 (Invitrogen, Carlsbad, CA, USA) according to manufacturer's instructions. Stable clones with the control plasmid or the NID1 expression vector were then selected in the presence of G418 (150 μg/ml), designated as OVCAR-3-vector and OVCAR-3-NID1-MC, respectively.

HEY cells were selected to generate cells with transient NID1 reduction. All siRNAs were chemically synthesized by Shanghai GenePharma (Shanghai, China). The sense sequences of the siRNA duplex included UUCUCCGAACGUGUCACGUUU (NC-siRNA), CAACGGAGCUUAUAACAUAUU (NID1-si798), GGAAAUACCAUGAGGAAGAUU (NID1-si2983). The blast data of NID1-siRNAs was supplied to address their specificity (seen in Supplementary Table 1). Transfection of HEY cells with siRNAs was performed using Lipofectamine RNAiMAX reagent (Invitrogen, Carlsbad, CA, USA) according to the manufacturer's instructions. Cells were then collected and subjected to analysis 72hr post-transfection.

### Cell treatment

To evaluate the function of ERK/MAPK signaling pathway in the EMT-promoting role of NID1, OVCAR-3-NID1-MC cells were treated with 50 μM U0126 (an effective MEK inhibitor, Cell Signaling Technology, Danvers, MA, USA) for 24h. These cells were lysed and subjected to Western blot analysis.

To examine the role of FAK in the activation of ERK/MAPK signaling pathway by NID1, OVCAR-3-NID1-MC cells were treated with 5 nM PF573228 (Sigma-Aldrich, St.Louis, Missouri, USA) for 24h, which effectively inhibited FAK phosphoryation on Tyr^397^. These cells were lysed and subjected to Western blot analysis.

### Quantitative RT-PCR

Total RNA was extracted from cultured cells using the Total RNA Kit I (Omega Bio-Tek, Doraville, GA, USA) according to the manufacturer's instructions. The cDNA was generated from 1 μg of total RNA using PrimeScript 1^st^ Strand cDNA Synthesis Kit (TaKaRa, Otsu, Japan) following the manufacturer's instructions. Quantitative real-time PCR was performed using the SYBR Premix Ex Taq^TM^ (Perfect Real Time) kit (TaKaRa, Otsu, Japan). The relative expression level of the target gene was calculated with the 2^−ΔΔCt^ method. The sequences of the primers used were provided in Supplementary Table 2.

### Western blotting

The standard Western blotting was conducted according to previously described procedures [38]. The information of the antibodies used were provided in Supplementary Table 3.

### Immunofluorescence staining

Cells were fixed and incubated with E-cadherin (20874-1-AP, Proteintech, Chicago, IL, USA) or Vimentin (5741, Cell Signaling Technology, Danvers, MA, USA) followed by the incubation with Alexa 594-conjugated anti-rabbit IgG (A-11012, Molecular Probes, Eugene, Oregon, USA). Cells were counterstained with 4′,6′-diamidino-2-phenylindole (Sigma-Aldrich, St.Louis, Missouri, USA) and examined by an Olympus BX51 fluorescence microscopy. The dilutions of these antibodies and reagents were provided in Supplementary Table 3.

### Cell proliferation assay

Cell proliferation was determined by Cell Counting Kit-8 (Beyotime, Jiangsu, China), according to the manufacturer's instructions. Ovarian cancer cells were seeded in 96-well plates at 0.2×10^4^ cells per well. Three independent experiments were performed in triplicates. The cell proliferation status at each time point was presented as the corresponding absorbance at 450nm.

### Wound healing assays

100×10^4^ cells were seeded in 60-mm culture dishes overnight in normal culture medium, and then the monolayer of the cells were scratched using a yellow pipette tip. After scraping, cells were incubated in low serum medium (2% FBS contained). Cells migrated across the wounds were photographed immediately and again at 1 day, 2 day, 3 day and 4 day after scrapping.

### Transwell migration and invasion assay

Transwell migration and invasion assay was conducted according to previously described procedures [38]. For transwell migration assay, 0.5×10^4^ cells were seeded, whereas 1×10^4^ cells were seeded for transwell invasion assay. Three independent experiments were performed in duplicates.

### Cell viability assay

Cytotoxicity assay was determined by Cell Counting Kit-8 (Beyotime, Jiangsu, China), according to the manufacturer's instructions. Ovarian cancer cells were seeded in 96-well plates at 1×10^4^ cells per well and then treated with the indicated concentration of cisplatin (Meilun Biotechnology, Liaoning, China) for 24h. Three independent experiments were performed in triplicates.

### Statistical analysis

Ovarian cancer microarray data were downloaded from the public GEO database (GSE53418, GSE53963, GSE26712 and GSE51373). All statistical analyses were carried out using the SPSS software, version 16.0 (SPSS INC, Chicago, IL, USA). Independent Student's t test was performed to analyze the significance of markers’ mRNA level, cell proliferation assay and cell viability assay. Paired Student's t test was applied in transwell migration/invasion assay. Overall survival curves were plotted by the Kaplan-Meier method and compared by log-rank test. Correlations between protein expression levels were evaluated with Pearson correlation test. All comparisons were two-tailed, and *P* values of <0.05 were taken to be statistically significant.

## SUPPLEMENTARY MATERIALS FIGURES AND TABLES



## Abbreviations

ECM: extracellular matrix
EMT: epithelial-mesenchymal transition
FAK: focal adhesion kinase
MET: mesenchymal-epithelial transition
NID1: nidogen-1

## References

[1] Torre LA, Bray F, Siegel RL, Ferlay J, Lortet-Tieulent J, Jemal A (2015). Global cancer statistics, 2012. CA Cancer J Clin.

[2] Chen W, Zheng R, Baade PD, Zhang S, Zeng H, Bray F, Jemal A, Yu XQ, He J (2016). Cancer statistics in China, 2015. CA Cancer J Clin.

[3] Yap TA, Carden CP, Kaye SB (2009). Beyond chemotherapy: targeted therapies in ovarian cancer. Nat Rev Cancer.

[4] Kang Y, Massagué J (2004). Epithelial-mesenchymal transitions: twist in development and metastasis. Cell.

[5] Nieto MA, Huang RY, Jackson RA, Thiery JP (2016). Emt: 2016. Cell.

[6] Thiery JP (2002). Epithelial-mesenchymal transitions in tumour progression. Nat Rev Cancer.

[7] Davidson B, Tropé CG, Reich R (2012). Epithelial-mesenchymal transition in ovarian carcinoma. Front Oncol.

[8] Vergara D, Merlot B, Lucot JP, Collinet P, Vinatier D, Fournier I, Salzet M (2010). Epithelial-mesenchymal transition in ovarian cancer. Cancer Lett.

[9] Kruegel J, Miosge N (2010). Basement membrane components are key players in specialized extracellular matrices. Cell Mol Life Sci.

[10] Yurchenco PD (2011). Basement membranes: cell scaffoldings and signaling platforms. Cold Spring Harb Perspect Biol.

[11] Timpl R, Brown JC (1996). Supramolecular assembly of basement membranes. BioEssays.

[12] Zhang Y, Xu B, Liu Y, Yao H, Lu N, Li B, Gao J, Guo S, Han N, Qi J, Zhang K, Cheng S, Wang H (2012). The ovarian cancer-derived secretory/releasing proteome: A repertoire of tumor markers. Proteomics.

[13] Li L, Zhang Y, Li N, Feng L, Yao H, Zhang R, Li B, Li X, Han N, Gao Y, Xiao T, Wu L (2015). Nidogen-1: a candidate biomarker for ovarian serous cancer. Jpn J Clin Oncol.

[14] Lee HK, Seo IA, Park HK, Park HT (2006). Identification of the basement membrane protein nidogen as a candidate ligand for tumor endothelial marker 7 in vitro and in vivo. FEBS Lett.

[15] Wu C, Chung AE (1991). Potential role of entactin in hemostasis. Specific interaction of entactin with fibrinogen A alpha and B beta chains. J Biol Chem.

[16] Baranowsky A, Mokkapati S, Bechtel M, Krügel J, Miosge N, Wickenhauser C, Smyth N, Nischt R (2010). Impaired wound healing in mice lacking the basement membrane protein nidogen 1. Matrix Biol.

[17] Pedrola N, Devis L, Llauradó M, Campoy I, Martinez-Garcia E, Garcia M, Muinelo-Romay L, Alonso-Alconada L, Abal M, Alameda F, Mancebo G, Carreras R, Castellví J (2015). Nidogen 1 and Nuclear Protein 1: novel targets of ETV5 transcription factor involved in endometrial cancer invasion. Clin Exp Metastasis.

[18] Beaufort CM, Helmijr JC, Piskorz AM, Hoogstraat M, Ruigrok-Ritstier K, Besselink N, Murtaza M, van Ĳcken WF, Heine AA, Smid M, Koudijs MJ, Brenton JD, Berns EM, Helleman J (2014). Ovarian cancer cell line panel (OCCP): clinical importance of in vitro morphological subtypes. PLoS One.

[19] Tothill RW, Tinker AV, George J, Brown R, Fox SB, Lade S, Johnson DS, Trivett MK, Etemadmoghadam D, Locandro B, Traficante N, Fereday S, Hung JA (2008). Novel molecular subtypes of serous and endometrioid ovarian cancer linked to clinical outcome. Clin Cancer Res.

[20] Bell D, Berchuck A, Birrer M, Chien J, Cramer DW, Dao F, Dhir R, DiSaia P, Gabra H, Glenn P, Godwin AK, Gross J, Hartmann L (2011). Integrated genomic analyses of ovarian carcinoma. Nature.

[21] Verhaak RG, Tamayo P, Yang JY, Hubbard D, Zhang H, Creighton CJ, Fereday S, Lawrence M, Carter SL, Mermel CH, Kostic AD, Etemadmoghadam D, Saksena G (2013). Prognostically relevant gene signatures of high-grade serous ovarian carcinoma. J Clin Invest.

[22] Bonome T, Levine DA, Shih J, Randonovich M, Pise-Masison CA, Bogomolniy F, Ozbun L, Brady J, Barrett JC, Boyd J, Birrer MJ (2008). A gene signature predicting for survival in suboptimally debulked patients with ovarian cancer. Cancer Res.

[23] Weiss MB, Abel EV, Mayberry MM, Basile KJ, Berger AC, Aplin AE (2012). TWIST1 is an ERK1/2 effector that promotes invasion and regulates MMP-1 expression in human melanoma cells. Cancer Res.

[24] Hong J, Zhou J, Fu J, He T, Qin J, Wang L, Liao L, Xu J (2011). Phosphorylation of serine 68 of Twist1 by MAPKs stabilizes Twist1 protein and promotes breast cancer cell invasiveness. Cancer Res.

[25] Xue G, Restuccia DF, Lan Q, Hynx D, Dirnhofer S, Hess D, Rüegg C, Hemmings BA (2012). Akt/PKB-mediated phosphorylation of Twist1 promotes tumor metastasis via mediating cross-talk between PI3K/Akt and TGF-β signaling axes. Cancer Discov.

[26] Hao L, Ha JR, Kuzel P, Garcia E, Persad S (2012). Cadherin switch from E- to N-cadherin in melanoma progression is regulated by the PI3K/PTEN pathway through Twist and Snail. Br J Dermatol.

[27] Koti M, Gooding RJ, Nuin P, Haslehurst A, Crane C, Weberpals J, Childs T, Bryson P, Dharsee M, Evans K, Feilotter HE, Park PC, Squire JA (2013). Identification of the IGF1/PI3K/NF κB/ERK gene signalling networks associated with chemotherapy resistance and treatment response in high-grade serous epithelial ovarian cancer. BMC Cancer.

[28] Tan TZ, Miow QH, Miki Y, Noda T, Mori S, Huang RY, Thiery JP (2014). Epithelial-mesenchymal transition spectrum quantification and its efficacy in deciphering survival and drug responses of cancer patients. EMBO Mol Med.

[29] Mitra SK, Schlaepfer DD (2006). Integrin-regulated FAK-Src signaling in normal and cancer cells. Curr Opin Cell Biol.

[30] Dedhar S, Jewell K, Rojiani M, Gray V (1992). The receptor for the basement membrane glycoprotein entactin is the integrin alpha 3/beta 1. J Biol Chem.

[31] Polyak K, Weinberg RA (2009). Transitions between epithelial and mesenchymal states: acquisition of malignant and stem cell traits. Nat Rev Cancer.

[32] Chen JY, Shen C, Yan Z, Brown DP, Wang M (2006). A systems biology case study of ovarian cancer drug resistance. Comput Syst Bioinformatics Conf.

[33] Bugide S, Gonugunta VK, Penugurti V, Malisetty VL, Vadlamudi RK, Manavathi B (2017). HPIP promotes epithelial-mesenchymal transition and cisplatin resistance in ovarian cancer cells through PI3K/AKT pathway activation. Cell Oncol (Dordr).

[34] Qin S, Li Y, Cao X, Du J, Huang X (2017). NANOG regulates epithelial-mesenchymal transition and chemoresistance in ovarian cancer. Biosci Rep.

[35] Liu S, Sun J, Cai B, Xi X, Yang L, Zhang Z, Feng Y, Sun Y (2016). NANOG regulates epithelial-mesenchymal transition and chemoresistance through activation of the STAT3 pathway in epithelial ovarian cancer. Tumour Biol.

[36] Chiu WT, Huang YF, Tsai HY, Chen CC, Chang CH, Huang SC, Hsu KF, Chou CY (2015). FOXM1 confers to epithelial-mesenchymal transition, stemness and chemoresistance in epithelial ovarian carcinoma cells. Oncotarget.

[37] Biddle A, Mackenzie IC (2012). Cancer stem cells and EMT in carcinoma. Cancer Metastasis Rev.

[38] Ji Y, Xie M, Lan H, Zhang Y, Long Y, Weng H, Li D, Cai W, Zhu H, Niu Y, Yang Z, Zhang C, Song F, Bu Y (2013). PRR11 is a novel gene implicated in cell cycle progression and lung cancer. Int J Biochem Cell Biol.

