# Supplementary material for: NID1, a new regulator of EMT required for metastasis and chemoresistance of ovarian cancer cells
Source: Oncotarget. 2017 Mar 13;8(20):33110–21. doi: 10.18632/oncotarget.16145 (PMC5464854; doi:10.18632/oncotarget.16145)
Supplement: Supplementary file 1 [file oncotarget-08-33110-s001.pdf]

# NID1, a new regulator of EMT required for metastasis and chemoresistance of ovarian cancer cells

## SUPPLEMENTARY FIGURES AND TABLES

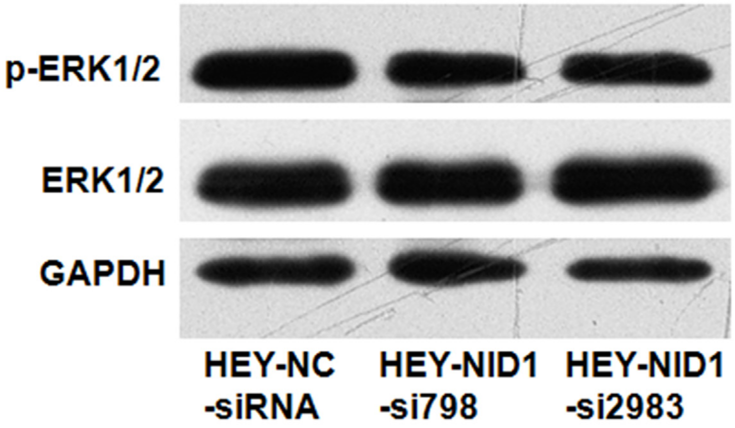


**Supplementary Figure 1: NID1 silencing in HEY cells reduced the level of phosphorylated ERK1/2.** Western blot for ERK1/2 and phosphorylated ERK1/2in the indicated cells, with GAPDH served as the loading control.

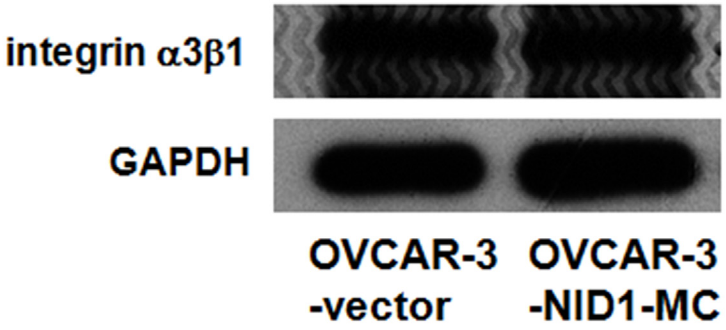


**Supplementary Figure 2: Expression of integrin α3β1 in OVCAR-3-NID1-MC cells.** Western blot for integrin α3β1 in total cell lysates of OVCAR-3-vector and OVCAR-3-NID1-MC cells. GAPDH served as an internal control of protein loading.

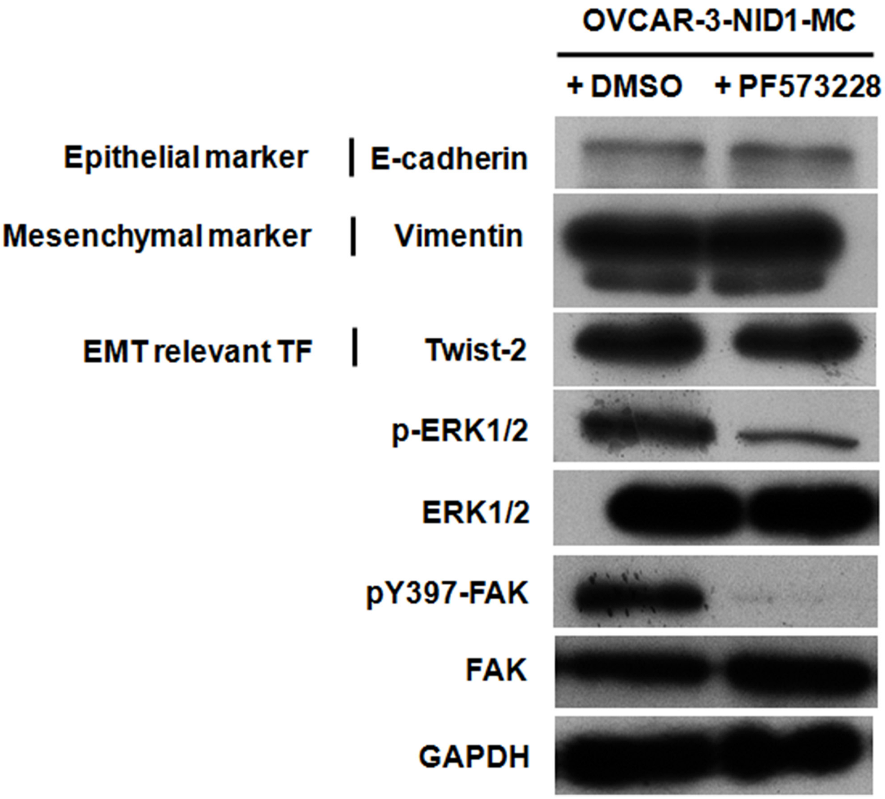


**Supplementary Figure 3: The NID1-induced activation of ERK/MAPK pathway was inhibited in the presence of PF573228 (an inhibitor for FAK phosphorylation on Tyr397).** Western blot for E-cadherin, Vimentin, Twist-2, phoshpo-ERK1/2, total ERK1/2, phospho-FAK and total FAK in total cell lysates of OVCAR-3-NID1-MC cells after PF573228 treatment. GAPDH served as an internal control of protein loading.

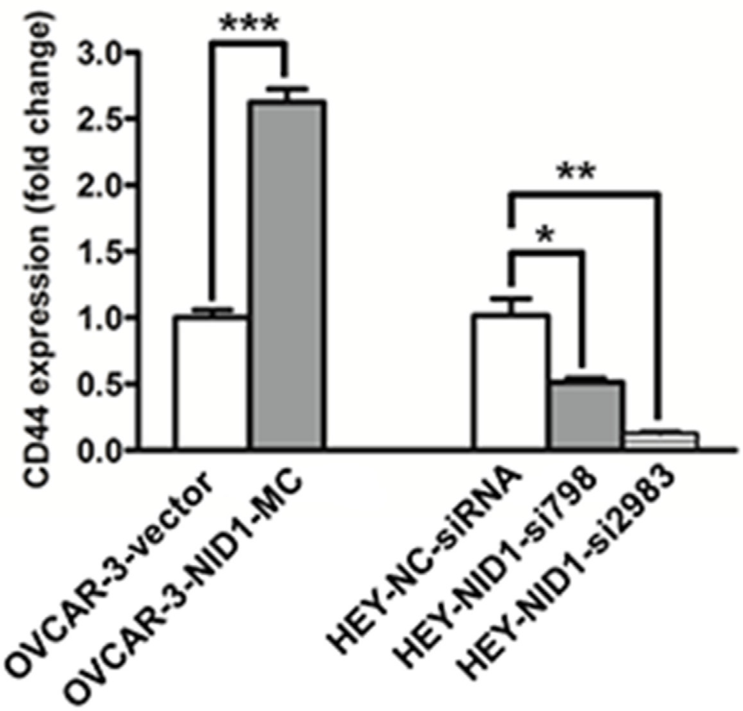


**Supplementary Figure 4: Expression of CD44 in OVCAR-3-NID1-MC cells and HEY cells transiently transfected with NID1-siRNA.** The CD44 mRNA levels in the indicated cells were detected by quantitative RT-PCR. Two independent experiments were performed in triplicates. Statistical analysis was done using two-sided independent Student's t test, $^{*}P<0.05$, $^{**}P<0.01$, $^{***}P<0.001$.

**Supplementary Table 1: The blast results of NID1-siRNAs**

| NID1-siRNA | Alignments | | | | | | |
|---|---|---|---|---|---|---|---|
| | Accession | Description | Max score | Total score | Query cover | E value | Ident |
| NID1-si798: 5'- CAACGGAGCUUA UAACAUAUU -3' | XM_0115441952 | PREDITED: Homo sapiens nidogen 1 (NID1) transcript variant X1, mRNA | 42.1 | 42.1 | 100% | 0.006 | 100% |
| | NM_0025082 | Homo sapiens nidogen 1 (NID1), mRNA | 42.1 | 42.1 | 100% | 0.006 | 100% |
| NID1-si2983: 5'- GGAAAUACCAU GAGGAAGAUU -3' | XM_0115441952 | PREDITED: Homo sapiens nidogen 1 (NID1) transcript variant X1, mRNA | 38.2 | 38.2 | 90% | 0.089 | 100% |
| | NM_0025082 | Homo sapiens nidogen 1 (NID1), mRNA | 38.2 | 38.2 | 90% | 0.089 | 100% |

**Supplementary Table 2: Primers used for RT-PCR analysis**

| Gene name* | Primer sequences |
|---|---|
| NID1 | F388:5'-TTATCCCCCTCCATCACTCA-3'<br>R539: 5'- CTCTTGCCTTTCTGGTCTGG-3' |
| CDH1 | F764: 5'-TGAAGGTGACAGAGCCTCTGGAT-3'<br>R915: 5'-TGGGTGAATTCGGGCTTGTT-3' |
| VIM | F1369: 5'-CCAAACTTTTCCTCCCTGAACC-3'<br>R1510: 5'-GTGATGCTGAGAAGTTTCGTTGA-3' |
| TWIST2 | F1246: 5'-TCTCCGTGATTGCTTGGCTA -3'<br>R1374: 5'-AGCAGGATACACAGCCACAC-3' |
| MDR1 | F1834: 5'-AGAGGGGATGGTCAGTGTTG -3'<br>R1947: 5'-GCTATCGTGGTGGCAAACAA-3' |
| ABCG2 | F2076: 5'-CAGCAGGTCAGAGTGTGGTT -3'<br>R2201: 5'-ACTGAAGCCATGACAGCCAA-3' |
| CD44 | F1657: 5'-GGCATGAGGGATATCGCCAA -3'<br>R1767: 5'-GGCTTGGTGTTGTCCTTCCT-3' |
| GAPDH | F833: 5′-ACC TGA CCT GCC GTC TAG AA-3′<br>R1060: 5′-TCC ACC ACC CTG TTG CTG TA-3′ |

*The protein products separately encoded by CDH1, CDH2 and VIM are E-cadherin, N-cadherin and Vimentin.

**Supplementary Table 3: Antibodies used in Western blotting and immunofluorescence staining**

| Protein name | Manufacture | Cat. number | Application (Working dilution) |
|---|---|---|---|
| GAPDH | Xianzhi Bio (Hangzhou, China) | AB-P-R 001 | IB (1:5000) |
| NID1 | R&D Systems (Minneapolis, MN, USA) | MAB2570 | IB (1:250) |
| E-cadherin | Proteintech (Chicago, IL, USA) | 20874-1-AP | IB (1:500), IF(1:100) |
| Vimentin | Cell Signaling Technology (Danvers, MA, USA) | 5741 | IB (1:1500), IF (1:150) |
| Twist-2 | MERCK Millipore (Billerica, MA, USA) | ABD24 | IB (1:3000) |
| ERK1/2 | Cell Signaling Technology (Danvers, MA, USA) | 4695 | IB (1:2000) |
| p-ERK1/2 | Cell Signaling Technology (Danvers, MA, USA) | 4370 | IB (1:2000) |
| JNK | Cell Signaling Technology (Danvers, MA, USA) | 9258 | IB (1:1000) |
| p-JNK | Cell Signaling Technology (Danvers, MA, USA) | 4668 | IB (1:1000) |
| p38 | Cell Signaling Technology (Danvers, MA, USA) | 8690 | IB (1:2000) |
| p-p38 | Cell Signaling Technology (Danvers, MA, USA) | 4511 | IB (1:2000) |
| FAK | Cell Signaling Technology (Danvers, MA, USA) | 3285 | IB (1:1000) |
| p-FAK (Tyr397) | Cell Signaling Technology (Danvers, MA, USA) | 8556 | IB (1:1000) |
| AKT1 | Epitomics (Burlingame, CA, USA) | 2957-1 | IB (1:3000) |
| p-AKT1 | Epitomics (Burlingame, CA, USA) | 2214-1 | IB (1:5000) |
| Integrin α3 | Ruiying Biological (Suzhou, China) | RLT2362 | IB (1:1000) |
| anti-Rabbit IgG | Molecular Probes (Eugene, Oregon, USA) | A-11012 | IF (1:1000) |
